# Single-Portal Proximal Biceps Tenodesis Using an All-Suture Anchor

**DOI:** 10.1016/j.eats.2021.11.023

**Published:** 2022-03-16

**Authors:** Malte Holschen, Benjamin Bockmann, Tobias L. Schulte, Kai-Axel Wit, Jörn Steinbeck

**Affiliations:** a Orthopedic Practice Clinic (OPPK), Münster; b Department for Orthopaedic Surgery, University Hospital, Bochum, Germany

## Abstract

The long head of the biceps is an important pain generator of the shoulder joint. Pathologies of the long head of the biceps involve superior labrum anterior to posterior lesions, pulley lesions, partial tears of the biceps tendon, biceps tendonitis, and medial biceps subluxation caused by full-thickness subscapularis tendon tears. Treatment of an inflamed or injured long head of the biceps by either tenotomy or tenodesis is often mandatory during shoulder arthroscopy to avoid persisting pain and possible revision procedures. In comparison with a tenotomy of the biceps tendon, a biceps tenodesis preserves the tension, anatomy, and cosmesis of the biceps muscle. The presented technique demonstrates a single portal technique for a proximal biceps tenodesis in the bicipital groove using an all-suture anchor.

Pathologies of the long head of the biceps (LHB) lead may result in pain and functional impairment of the shoulder joint.^1^ Besides sole biceps tendonitis, injuries, or degenerative lesions like SLAP lesions, partial tears of the LHB also can lead to lead pain and inflammation.^2^, ^3^, ^4^ Pulley lesions and adjacent rotator cuff tears, especially those of the subscapularis tendon, lead to instability of the LHB in the rotator interval.^5^^,^^6^ Over the time the unstable LHB degenerates and loses its initial round structure to change to a flat and thick structure (hourglass phenomenon).^7^ Additional degenerative changes of the cartilage below an unstable biceps tendon have been described.^8^ Ongoing degeneration may finally end in partial or complete tears of the LHB.^9^ Overhead athletes are often affected by pathologies of the LHB.^10^

During rotator cuff repair, management of the LHB by tenotomy or tenodesis should be considered to avoid pain and stiffness caused by postoperative biceps tendonitis.^11^ Rotator cuff repair may lead to pain and stiffness caused by chronic biceps tendonitis due to an altered anatomy of the rotator interval or irritating sutures in the supraspinatus or subscapularis tendon. In particular, larger rotator cuff tears may require additional biceps tenotomy or tenodesis to protect the repaired rotator cuff from an unstable biceps tendon.

Several possibilities for management of these LHB lesions exist.^11^^,^^12^ The easiest and fastest way is a tenotomy of the LHB at the supraglenoid tubercle. However, biceps tenotomy is related to certain potential disadvantages:

• the biceps muscle belly may drop distally and become more prominent (Popeye sign)^13^;
• potential biceps muscle cramping may occur^13^; and
• reduced power during forearm supination may occur.^13^

The presented technique avoids these disadvantages, because the LHB is fixed to the most proximal entrance of the bicipital groove and thus protected from moving distally. In comparison with other techniques used for biceps tenodesis like suprapectoral biceps tenodesis in the middle or the inferior part of the bicipital groove or subpectoral tenodesis, the presented technique is probably the fastest and the easiest way to prevent the LHB from moving distally.

The whole procedure can be performed under direct intra-articular visualization. Next to the posterior viewing portal, only one more anterolateral portal is mandatory to perform the tenodesis. In comparison with standard rotator cuff anchors or biceps tenodesis screws, the presented technique works with an all-suture anchor, which is easy to apply and which does neither require large implants or large drill holes.

## Surgical Technique (With Video Illustration)

### Patient Positioning and Anesthesia

The patient is placed in a beach-chair position on a standard surgical table. The affected arm is supported in an arm holder (TRIMANO FORTIS; Arthrex, Naples, FL) in neutral forward flexion and rotation. The operation is conducted with the patient under general anesthesia and an additional interscalene brachial plexus block. The systolic blood pressure is kept below a systolic maximum of 100 mm Hg to prevent excessive bleeding.

### Intra-Articular Assessment of the Biceps Tendon

The arthroscope is introduced through a standard posterior portal and the joint is assessed systematically. Special regard is given to rotator interval lesions, respectively pulley lesions, chondral defects located on the humeral head below the LHB, SLAP lesions, anterior-superior rotator cuff tears, and biceps tendonitis. In cases of rotator cuff tears located closely to the LHB (supraspinatus tendon tear or subscapularis tendon tear), the stability of the LHB is assessed by internal and external rotation of the arm. A probe inserted through the rotator interval or through an anterior portal may be helpful to visualize medial or posterior instability of the LHB. Medial instability occurs in cases of subscapularis tendon tears, whereas posterior instability is caused by supraspinatus tendon tears. An arthroscopic grasper may be helpful to pull the LHB out of the bicipital groove to confirm biceps tendonitis. In cases of a wide canal, the arthroscope can be moved into the proximal sulcus of the LHB to confirm synovitis and inflammation.

### Biceps Tenodesis

**Fig 1:**
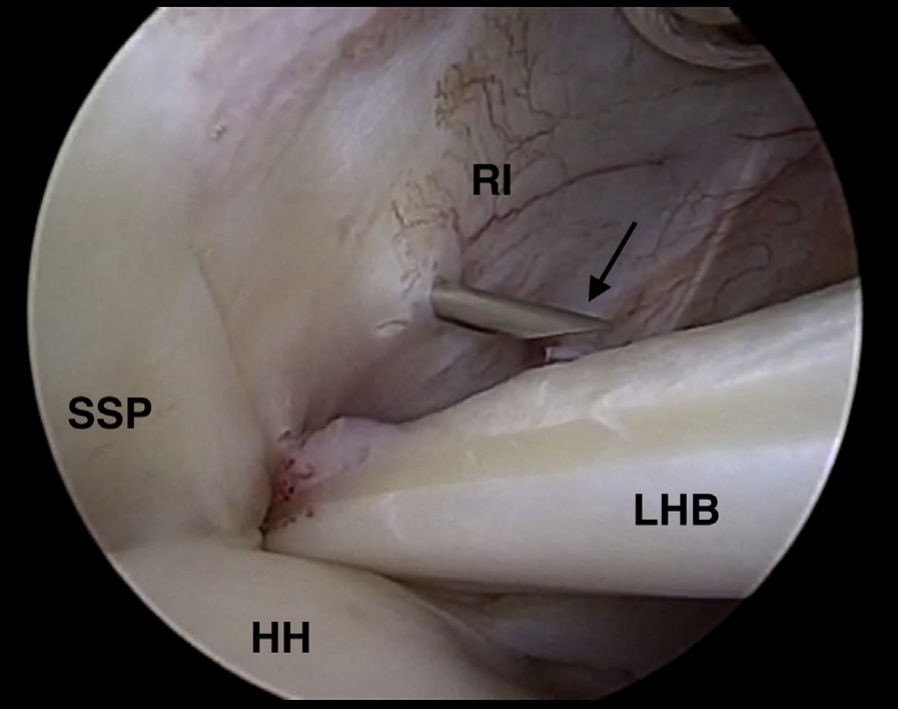
Arthroscopic view of a left shoulder in the beach-chair position. The arthroscope is located in a standard posterior viewing portal. The rotator interval (RI) is located with a spinal needle (black arrow) in an outside-in fashion above the long head of the biceps tendon (LHB) through an anterolateral portal. The image also shows the supraspinatus tendon (SSP) and the superior aspect of the humeral head (HH).

When indicated, suprapectoral intra-articular tenodesis of the LHB is performed through a 1.5-cm anterolateral portal. The rotator interval is located with a spinal needle in an outside-in fashion, whereas the arthroscope is used to visualize both the rotator interval and the LHB (Fig 1). The needle should penetrate the skin and rotator interval in line with the LHB. After confirmation of correct needle positioning, a number 11 scalpel is used for skin incision as well as sufficient incision of the rotator interval. The incision of the rotator interval should include the most proximal centimeter of the entrance into the bicipital canal. If skin incision or incision of the rotator interval are too small, the LHB may get lost during extraction above skin level.

**Fig 2:**
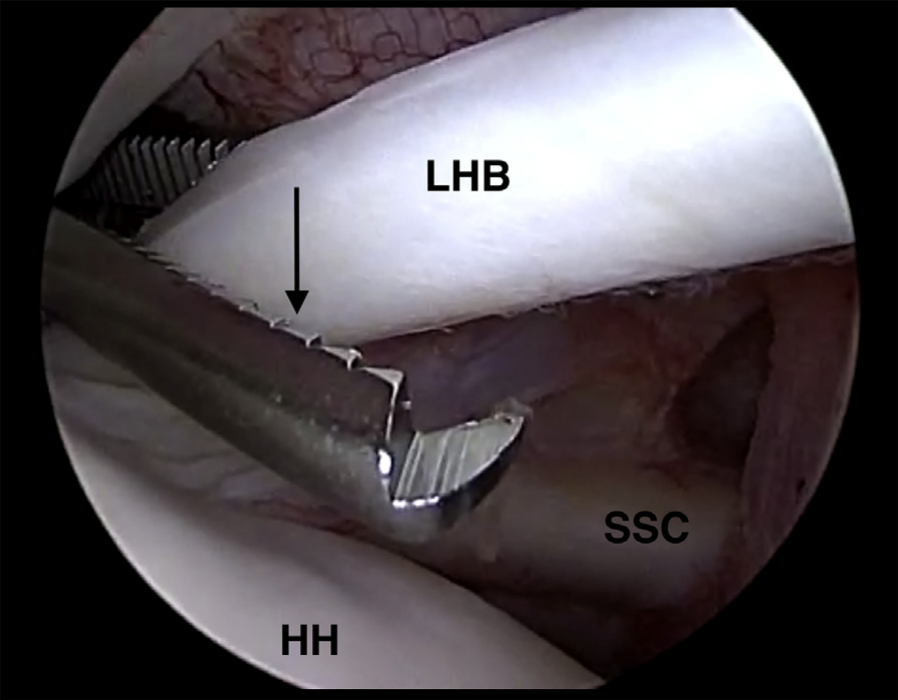
Arthroscopic view of a left shoulder in the beach-chair position. The arthroscope is located in a standard posterior viewing portal. The long head of the biceps tendon (LHB) tendon is grasped with a Kocher clamp (black arrow) inserted through the anterolateral portal prior to biceps tenotomy. The subscapularis tendon (SSC) is located anteriorly to the humeral head (HH).

**Fig 3:**
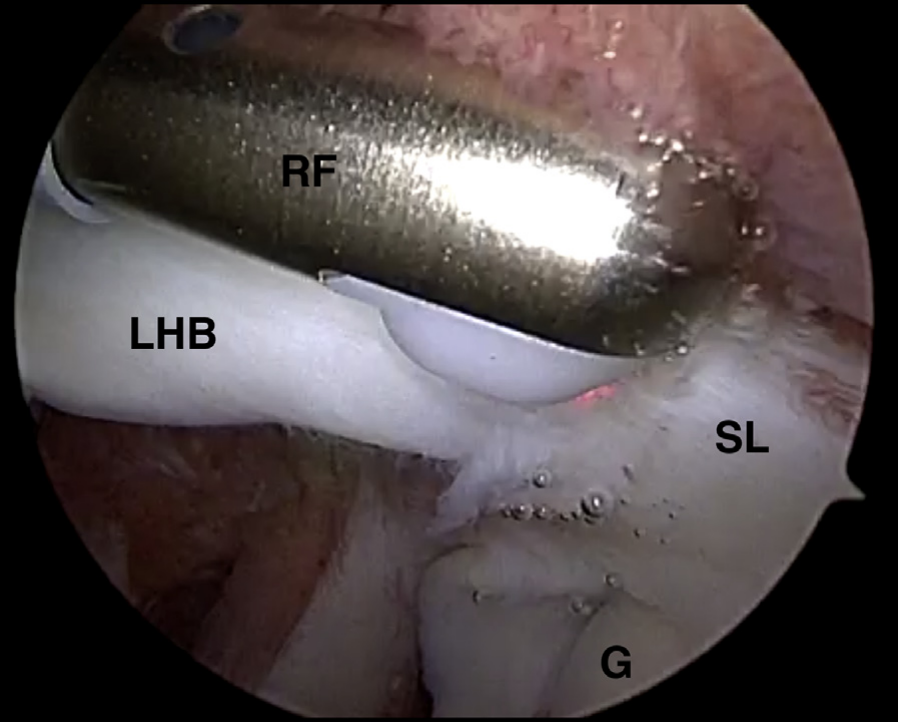
Arthroscopic view of a left shoulder in the beach-chair position. The arthroscope is located in a standard posterior viewing portal. The radiofrequency device (RF) is inserted through the anterolateral portal to carry out biceps tenotomy at the superior labrum (SL), immediately above the glenoid (G). (LHB, long head of the biceps tendon.)

**Fig 4:**
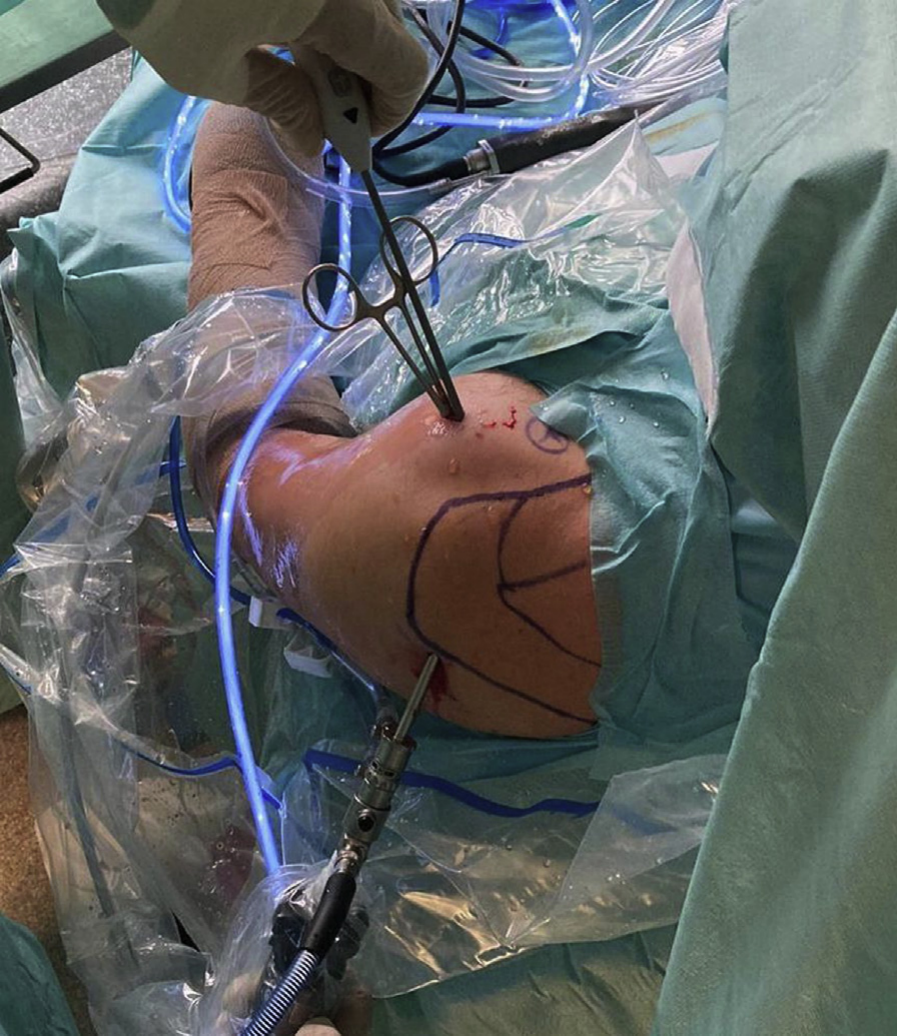
Extra-articular view of a left shoulder in the beach-chair position from lateral during biceps tenotomy using a radiofrequency device and a Kocher clamp through a single anterolateral portal.

The biceps tendon needs to be secured with a Kocher clamp through the anterolateral portal (Fig 2). Subsequently, LHB tenotomy is carried out right at the attachment on the superior labrum through the very same portal using a radiofrequency device (ApolloRF; Arthrex) (Figs 3 and 4). After complete tenotomy, the LHB is pulled out of the anterolateral portal. Care must be taken to avoid losing the biceps tendon during this step, because it may potentially get lost in the bicipital canal. If the LHB gets lost, tenotomy or subpectoral tenodesis should be considered.

**Fig 5:**
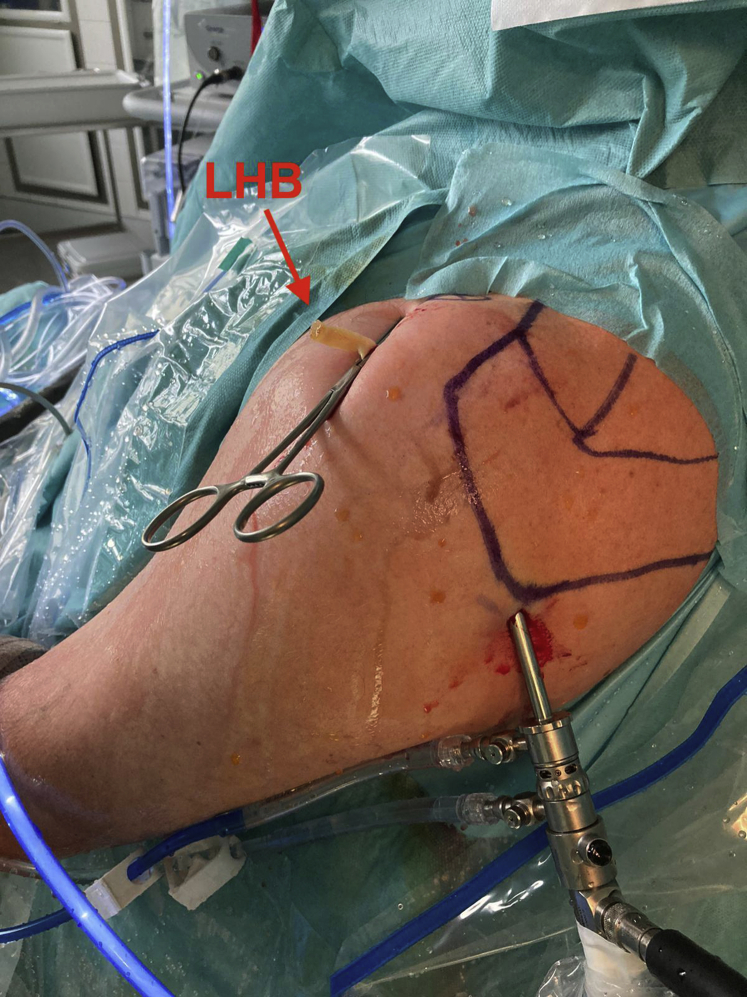
Extra-articular view of a left shoulder in beach chair position from lateral after biceps tenotomy. The long head of the biceps (LHB) is pulled out of the anterolateral portal and subsequently secured with a Kocher clamp above skin level.

After pulling the LHB out of the anterolateral portal, it is secured with a second Kocher clamp above skin level and shortened by 2 cm (Fig 5). The proximal portion of the bicipital groove needs to be debrided with a shaver or a burr to create a bleeding bed. The cortical bone should not be removed entirely during this step to avoid pulling out of the suture anchor.^14^ If mandatory, a radiofrequency device may be inserted to perform synovectomy in the bicipital groove and in the rotator interval region to avoid postoperative pain.

**Fig 6:**
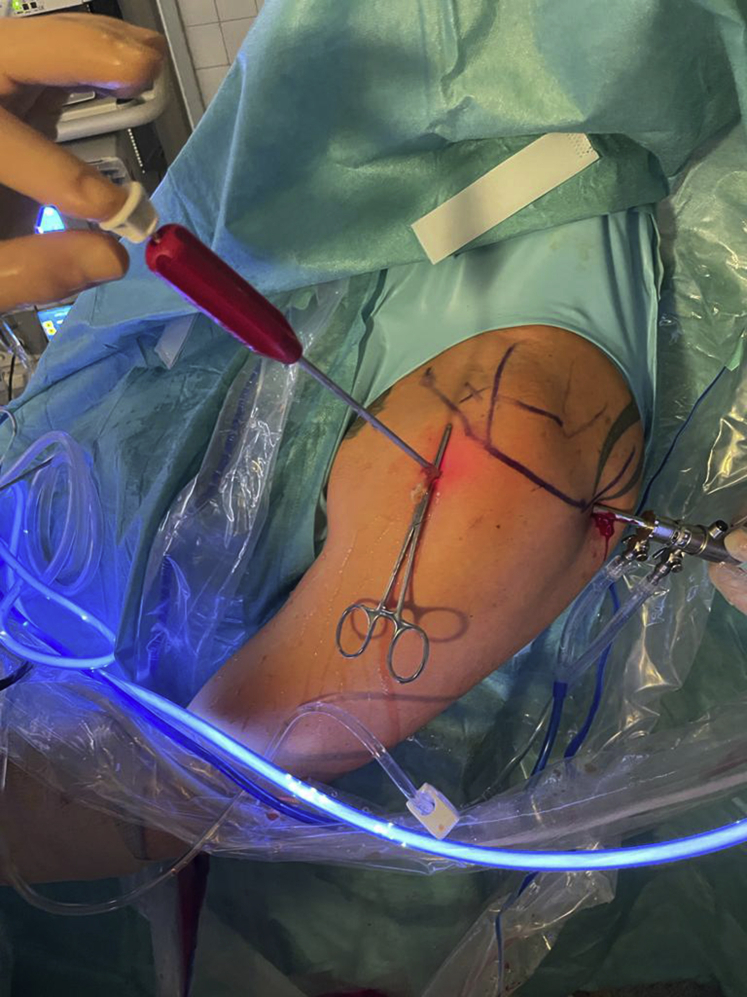
Extra-articular view of a left shoulder in beach chair position from lateral. A spear with a blunt tip obturator is inserted through the anterolateral portal, while the biceps tendon is secured with a Kocher clamp.

**Fig 7:**
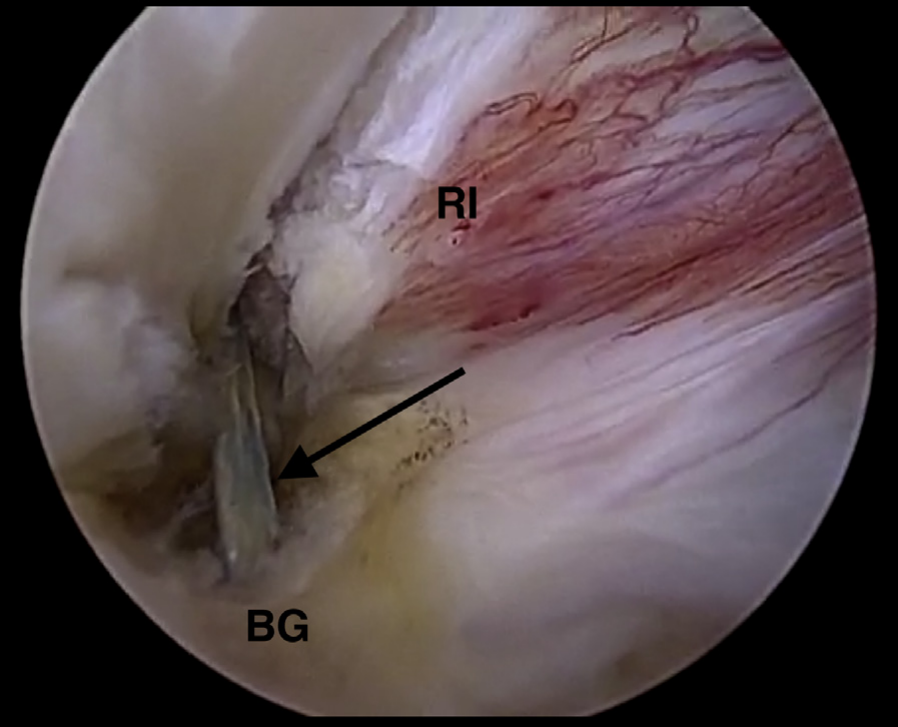
Intra-articular view of an all-suture anchor and its suture tails (black arrow) in the bicipital groove (BG) below the rotator interval (RI). The image shows a left shoulder. The arthroscope is located in a standard posterior viewing portal. The anchor was inserted through an anterolateral portal.

**Fig 8:**
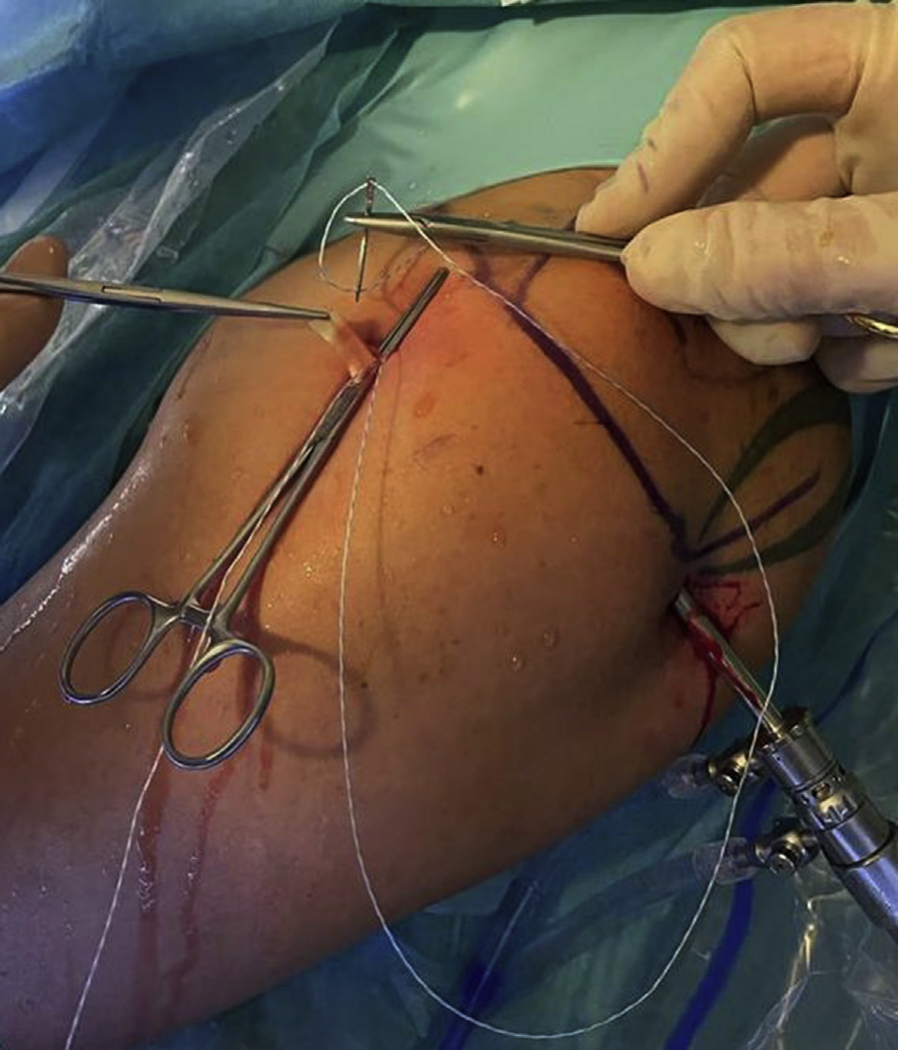
Extra-articular view of a left shoulder in beach chair position from lateral. One limb of the all-suture anchors is employed for arming of the biceps tendon.

Subsequently, a spear with a blunt tip obturator is inserted through the anterolateral portal (Fig 6). After perpendicular placement onto the bone bed of the bicipital groove, the obturator is removed and a 1.6-mm depth stop drill is introduced to create a bone socket. After removal of the drill, an all-suture anchor (FiberTak Soft Anchor; Arthrex) is impacted into the bone socket with an inserter handle. By pulling the inserter handle with the sutures, the anchor is set into the bone (Fig 7). Two suture limbs of the all-suture anchor (FiberWire #2; Arthrex) remain above skin level after spear and inserter handle have been removed. One of the sutures is used to arm the LHB with Krackow stitches using a standard surgical needle (Fig 8). The Kocher clamp needs to be removed after this step.

**Fig 9:**
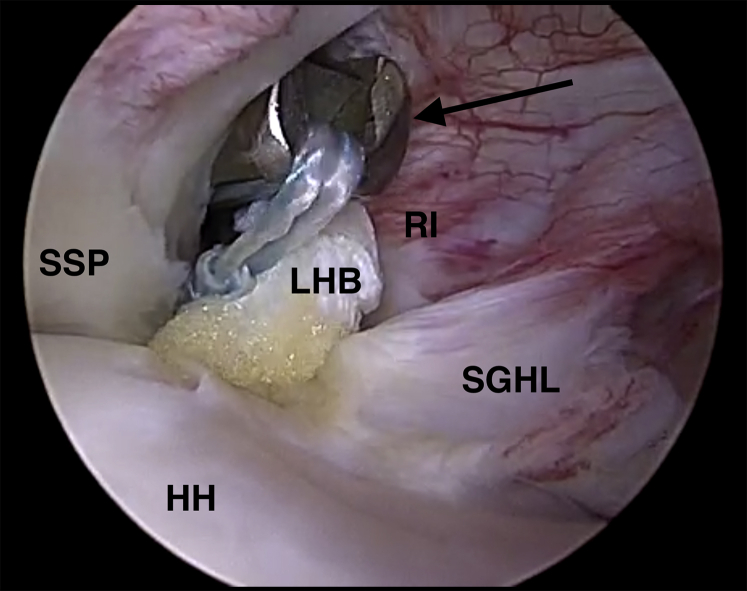
Final view of the completed suprapectoral tenodesis of the long head of the biceps (LHB) during cutting the sutures after knot tying (black arrow). The LHB is fixed to the entrance of the bicipital groove in the direct neighborhood of the rotator interval (RI), the superior glenohumeral ligament (SGHL), the humeral head (HH), and the supraspinatus tendon (SSP). The image shows a left shoulder. The arthroscope is located in a standard posterior viewing portal. The suture cutter was inserted through an anterolateral portal.

By pulling the free suture limb, which has not been used for tendon arming, the LHB is fixed to the bone bed. The pulling suture is used as the post for knot-tying with 7 alternating half hitches. The tendon is visualized through the posterior portal during knot tying. A suture cutter is employed to cut the sutures 0.5 cm above the last knot under arthroscopic control (Fig 9). The described technique is presented in Video 1.

A probe inserted through the anterolateral portal confirms the correct tension of the LHB. If no further procedures like rotator cuff repair or subacromial decompression are mandatory, the procedure may be finished with standard skin closure and wound dressing.

### Postoperative Rehabilitation

The affected shoulder is immobilized in an abduction sling for 2 weeks. For the first 2 weeks, passive exercises of the shoulder and the elbow are permitted. After the sling has been removed, the patient starts assisted active movements with unlimited range of motion. For the first 6 postoperative weeks, active elbow flexion or forearm supination against resistance are not permitted. After 6 weeks, progressive active exercises against resistance for the shoulder and the elbow are allowed. Return to contact sports or weight-lifting is possible after three months.

## Discussion

For arthroscopic shoulder surgeons, biceps tenodesis is an important procedure to avoid persistent pain and instability after a surgical procedure.^6^ In particular, younger and active patients should be treated by biceps tenodesis rather than by biceps tenotomy to avoid impaired cosmesis of the upper arm and potentially impaired forearm supination and elbow flexion force.^15^^,^^16^ Some biceps tenodesis techniques are demanding and time-consuming or require larger implants like interference screws.^17^^,^^18^ The presented technique introduces a fast and reproducible way to perform a proximal biceps tenodesis through a single anterolateral portal with an all-suture anchor.

Many other techniques for biceps tenodesis have been described.^19^^,^^20^ All types of suprapectoral tenodesis share a risk for persistent pain in the bicipital groove because of ongoing synovitis, muscle cramping, and mechanical irritation of this sensitive area, which may be a reason for revision procedures.^20^^,^^21^ Some all-arthroscopic techniques avoid pulling the LHB above skin level. This may be beneficial to avoid postoperative infection caused by contact with the skin. However, infections after suprapectoral biceps tenodesis are very rare.^12^ Pulling the LHB above the skin level is advantageous, because tendon arming is facilitated and more stable (Krackow stitches). In addition, all-arthroscopic suprapectoral tenodesis techniques, in particular when performed under extra-articular visualization, are surgically more demanding and less reproducible for unexperienced shoulder surgeons.

Subpectoral biceps tenodesis has its own advantages and disadvantages. A relevant clinical difference between suprapectoral and subpectoral biceps tenodesis has not been observed in a recent literature review.^22^ The most important advantage is that the LHB is completely removed from its canal and the area underneath the insertion of the pectoralis major tendon. In patients with significant tenderness along the inferior part of the bicipital canal and the pectoralis major insertion during physical examination, subpectoral biceps tenodesis may be beneficial.^23^ However, this technique has some disadvantages: There is an increased risk for neurovascular injuries in the axillary fold and an increased risk for infection.^24^ If larger drill tunnels are established in the proximal diaphysis of the humerus, there is also a risk for humeral shaft fractures.^25^

Advantages and disadvantages of the described technique for suprapectoral tenodesis using an all-suture anchor are summarized in Table 1. Although our presented technique is easy to apply and reproducible, it is related to certain risks like losing the biceps tendon in the bicipital canal and for displacement of the all-suture anchor. It is important to create an anterolateral portal, which is located directly above the biceps tendon in the proximal part of its canal and which does not involve the anterosuperior rotator cuff. If the incisions are too small, the biceps tendon may get lost during extraction above skin level. Thus, it is crucial to secure the tendon with the tip of a Kocher clamp under arthroscopic visualization before the tenotomy is carried out. After tenotomy, gentle pulling and rotation of the clamp will facilitate extracting the LHB above skin level. Then, it is very important to use a second Kocher clamp to grab the most distal part of the tendon above the skin level with the proximal part of the clamp. This makes sure, that the construct is temporarily fixed above skin level to perform subsequent anchor placement and tendon arming.

**Table 1:** Advantages and Disadvantages of a Single-Portal Proximal Biceps Tenodesis Using an All-Suture Anchor

Advantages	Disadvantages
Fast procedure Only one working portal Small implant Easy visualization	The biceps tendon remains in the groove Secure knot-tying is required The biceps tendon needs to be pulled out above skin level (potential contamination)

As mentioned, the shaver should not remove the whole cortical bone in the proximal bicipital groove to guarantee optimal stability of the all-suture anchor. In cases of poor spongious bone, the anchor may pull out during tensioning or knot tying. If the anchor is pulled out, the LHB can be fixed with a subpectoral tenodesis or it may be fixed with a knotless suture anchor in the bicipital groove. For this procedure, the LHB is pulled above skin level with the 2 suture limbs and secured with a Kocher clamp. The sutures and the anchor are removed from the LHB before a nonabsorbable suture is applied with Krackow stitches. After tapping a hole into the proximal bicipital groove, the LHB is fixed with a knotless suture anchor under arthroscopic visualization.

It is important to control the mobility of the 2 suture limbs in the anchor eyelet prior to tendon arming. After tendon arming the other suture limb is used to pull the LHB onto the bone bed in the bicipital groove. This step needs to be visualized carefully to make sure that the tendon does not get trapped above the rotator interval. A switching stick may be helpful to gently manipulate the LHB, if the tendon is trapped above the rotator interval. This problem does not occur, if the incisions are large enough.

Pearls and pitfalls are summarized in Table 2. The main advantage of the described technique is a fast and easy fixation of the LHB in the proximal bicipital groove using a small implant. Next to the described intraoperative risks like losing the tendon after tenotomy or dislocation of the suture anchor, persistent pain in the bicipital canal is a major concern. Usually, these symptoms will subside after a while. However, patients need to be informed about this risk before the surgical procedure.

**Table 2:** Pearls and Pitfalls of a Single-Portal Proximal Biceps Tenodesis Using an All-Suture Anchor

Pearls	Pitfalls
A Kocher clamp grabs the mid-portion of the intra-articular biceps tendon with its tip A radiofrequency device may be used for synovectomy in the proximal bicipital groove The superior labrum should be preserved during biceps tenotomy Slight forward elevation of the arm aids visualization of the bicipital groove Gentle pulling and rotation of the Kocher clamp facilitates pulling the biceps tendon above skin level A large anterolateral portal involving the rotator interval is useful to pull the biceps tendon above skin level	The biceps tendon may get lost during pulling it out above skin level Degenerated and thickened biceps tendons may tear or get lost during the pull-out procedure Poor visualization may lead to malpositioning and displacement of the all-suture anchor Weakening of the cortical bone in the bicipital groove may results in dislocation of the all-suture anchor Biceps tenotomy with a radiofrequency device and a Kocher clamp in the same portal may be demanding Insufficient knot-tying may lead to early failure

The describe technique should not be performed in cases of poor bone quality and poor quality of the LHB, especially in elderly patients. These patients should be considered for biceps tenotomy. If the LHB has become very thick during a yearlong degenerative process, suprapectoral tenodesis may be beneficial, because pulling the tendon out of its canal during subpectoral tenodesis may be impossible and likely leads to tearing of the distal portion of the tendon. A summary of advantages, risks and limitations is shown in Table 3.

**Table 3:** Advantages, Risks, and Limitations of a Single-Portal Proximal Biceps Tenodesis Using an All-Suture Anchor

Advantages	Risks	Limitations
Easy to learn and to apply Optimized visualization of relevant anatomical structures (bicipital groove, biceps tendon, rotator interval) Secure anchor placement Strong fixation construct	Anchor dislocation Dislocation of the biceps tendon Persistent pain in the bicipital groove Shoulder stiffness	Poor tissue quality of the biceps tendon may lead to early failure Poor bone quality in the bicipital groove may lead to anchor dislocation Pulling the tendon above skin level may hindered in obese patients This technique is inapplicable in cases of a torn biceps tendon

## Supplementary Data

ICMJE author disclosure forms

Video 1 The video demonstrates the single-portal proximal biceps tenodesis using an all-suture anchor. The video demonstrates the left shoulder of a patient in the beach-chair position. The arthroscope is introduced through a standard posterior viewing portal throughout the procedure. Instrument are introduced through an anterolateral portal involving the rotator interval.
